# The effect of *Haemophilus influenzae* type B and pneumococcal conjugate vaccines on childhood meningitis mortality: a systematic review

**DOI:** 10.1186/1471-2458-13-S3-S21

**Published:** 2013-09-17

**Authors:** Stephanie Davis, Daniel Feikin, Hope L Johnson

**Affiliations:** 1 International Vaccine Access Center, Department of International Health, Johns Hopkins Bloomberg School of Public Health, 855 Wolfe St, Suite 600, Baltimore, MD, USA

## Abstract

**Background:**

Two of the most prevalent causes of severe bacterial meningitis in children, *Haemophilus influenzae* type B (Hib) and *Streptococcus pneumoniae*, are preventable by existing vaccines increasingly available in developing countries. Our objective was to estimate the dose-specific effect of Hib and pneumococcal conjugate vaccines (PCV) on childhood meningitis mortality in low-income countries for use in the Lives Saved Tool (LiST).

**Methods:**

We systematically searched and reviewed published vaccine efficacy trials and observational studies reporting the effect of Hib or PCV vaccines on organism-specific meningitis, bacterial meningitis and all-cause meningitis incidence and mortality among children less than five years old in low- and middle-income countries. Data collection and quality assessments were performed using standardized guidelines. For outcomes available across multiple studies (≥2) and approximating meningitis mortality, we pooled estimates reporting dose-specific effects using random effects meta-analytic methods, then combined these with meningitis etiology data to determine the preventable fraction of childhood meningitis mortality for inclusion in LiST.

**Results:**

We identified 18 studies of Hib conjugate vaccines reporting relevant meningitis morbidity and mortality outcomes (2 randomized controlled trials [RCTs], 16 observational studies) but few provided dose-specific effects. A meta-analysis of four case-control studies examined the dose-specific effect of Hib conjugate vaccines on Hib meningitis morbidity (1 dose: RR=0.64, 95% CI 0.38-1.06; 2 doses: RR=0.09, 95% CI 0.03-0.27; 3 doses: RR=0.06, 95% CI 0.02-0.22), consistent with results from single RCTs. Pooled estimates of two RCTs provided evidence for the effect of three doses of PCV on vaccine-serotype meningitis morbidity (RR=0.16, 95% CI 0.02-1.20). We considered these outcomes of severe disease as proxy estimates for meningitis mortality and combined the estimates of protective effects with meningitis etiology data to provide an estimate of the preventable fraction of childhood meningitis mortality with three doses of Hib (38-43%) and pneumococcal conjugate vaccines (28-35%) for use in LiST.

**Conclusions:**

Few RCTs or vaccine effectiveness studies evaluated the dose-specific impact of Hib and PCV vaccines on childhood meningitis mortality, necessitating use of proxy measures to estimate population impact in LiST. Our analysis indicates that approximately three-quarters of meningitis deaths are preventable with existing Hib and PCV vaccines.

## Background

Meningitis accounts for an estimated 180,000 deaths every year in children less than five years of age globally; and approximately 20% of survivors experience long-term disabling sequelae [1,2]. Bacterial pathogens are responsible for the majority of severe pediatric meningitis outside the neonatal period, and many cases are preventable with existing conjugate vaccines protective against *Haemophilus influenzae* type B (Hib) and *Streptococcus pneumoniae* (pneumococcus) forms of meningitis [3,4].

Although Hib and pneumococcal vaccines have been available for more than a decade in developed countries, they have only recently been made available to children in low-income countries through financial support from the GAVI Alliance. As of December 2012, nearly all low-income GAVI-eligible countries (70/73) now have Hib conjugate vaccine included in their national immunization programs. The first GAVI-eligible country introduced pneumococcal conjugate vaccine (PCV) in 2009. Since 2009, 23 other countries have introduced PCV and 26 have approval for GAVI-supported PCV introductions over the next 2 years [5,6].

Understanding the effectiveness of Hib and PCV vaccines remains critical to inform decision-making for the remaining low- and middle-income countries yet to introduce these vaccines into their national immunization programs. Use of the existing evidence on the effectiveness of Hib and PCV vaccines can also inform input parameters for mathematical models such as the Lives Saved Tool (LiST) [7], developed by the Child Health Epidemiology Research Group (CHERG), to project the number of lives saved by scaling up use of these vaccines in the context of other child survival interventions, providing important information for program planning.

In the absence of an existing estimate for meningitis mortality, LiST currently uses estimates of the effectiveness of Hib and PCV against childhood pneumonia morbidity as a proxy. Here we present a systematic review and meta-analysis on the effects of Hib and PCV vaccines in prevention of childhood meningitis mortality, with the intent to update parameters used in LiST to model the dose-specific effectiveness of these vaccines using consistent and standard methods according to CHERG guidelines [8].

## Methods

### Study identification, selection and abstraction

**Figure 1:**
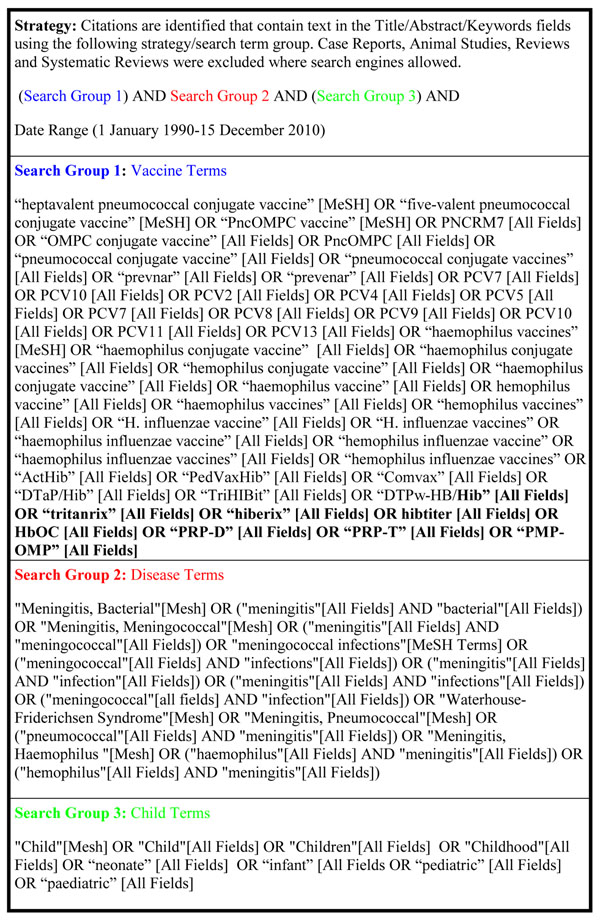
PubMed literature search terms

We conducted a systematic search of the published literature to identify both trials and observational studies reporting on the effect of Hib or PCV vaccines on meningitis-related morbidity and mortality outcomes among children less than five years old in low- and middle-income countries [9] published between 1990 and December 2010. Search terms included various combinations of terms for pneumococcal vaccine, *Haemophilus* vaccine, and meningitis and used controlled vocabulary where possible (Figure 1). Databases searched include Pubmed, Embase, Web of Science, Scopus, Africa-Wide Information, Global Health, Cochrane, and the WHO Library database (WHOLIS) and WHO regional databases (Index Medicus for the Eastern Mediterranean Region, Western Pacific Region Index Medicus, African Index Medicus, IndMed, PAHO Virtual Health Library, and Latin America and Caribbean Health Sciences Literature (LILACS). We also searched the System for Information on Grey Literature in Europe (SIGLE) and websites including http://ClinicalTrials.gov, Hibaction, PneumoADIP, and the World Health Organization Hib and Pneumococcus for relevant citations [4,10,11].

All studies with data from only high-income countries were excluded. We double-reviewed the titles and abstracts of the remaining citations. The full-text of included citations was retrieved where possible and double-reviewed for inclusion with discrepancies resolved by consensus between reviewers. We excluded animal, in-vitro and immunogenicity studies, those without primary data on outcomes of interest, time-series observational studies with only post-vaccine introduction data, and studies conducted among vulnerable populations not representative of the general population. We included randomized controlled trials (RCTs), cohort and observational studies (case-control and pre- vs. post-vaccine introduction time periods) that assessed use of Hib or PCV vaccines and the effect on Hib or PCV-specific meningitis, bacterial or probable bacterial meningitis according to authors’ case definition (typically, according to WHO criteria on CSF findings [12]), or all-cause meningitis incidence or mortality or all-cause mortality among children less than five years of age in low-or middle-income countries, if the study design included a control group (or time period in the instance of pre- vs. post vaccine introduction time-series studies) and standard error information was available. For studies evaluating the effectiveness of PCVs, we also abstracted vaccine serotype-specific (VT) and all-serotype (AT) outcomes. Foreign language articles were reviewed by proficient abstractors or translated where possible. In instances where the same outcome was reported from multiple citations studying the same or overlapping study populations, we selected only the publication with the most complete or recent results for inclusion.

We double-abstracted information about the study location, design, population characteristics and size, type of vaccine, and immunization coverage directly into a customized Microsoft Access database (Microsoft). All included studies were independently abstracted and harmonized by consensus, then re- confirmed by a senior adjudicator. For outcomes of interest we abstracted information on the number of events in the control (or pre-introduction) and intervention (or post-introduction) groups, effect measures (risk ratios, RR) and standard error information by numbers of doses of vaccine administered. All effect estimates were converted into RR where otherwise presented and 95% confidence intervals calculated from available information in the publication and population data from national [13] and United Nations [14] statistical sources when necessary.

For pre- vs. post-vaccine introduction time-series studies, the effect measure was typically calculated by comparing number of events occurring in the last year prior to vaccine introduction (pre-introduction) to number of events in the last year for which data are available post-vaccine introduction (post-introduction). When available, immunization coverage data informed categorization of years into these time periods. Results were sometimes available from the same setting for both pre- vs. post- introduction and nested case-control study designs, and in these instances the case-control data were used in the analysis. For RCTs, age of the study population indicates age at time of enrollment recruitment.

### Quality assessment and effect size estimates for LiST

We assessed the generalizability and quality of evidence using the CHERG scoring system [8]; the Cochrane Collaboration’s GRADE criteria [15] for determining risk of bias were used to guide score assignment for RCTs; and the Newcastle-Ottawa Quality Assessment Scale [16] was used to inform quality assessment for non-randomized studies in meta-analyses. Due to the severity of meningitis, in which virtually all diagnosed cases are hospitalized, population generalizability was not downgraded for studies ascertaining only hospitalized cases. Details of study data quality scoring, including inclusion in relevant Cochrane reviews [10,11] are presented in Supplementary Tables 1 and 2 in Additional File 1.

We log-transformed study effect measures (RR), stratified analyses by study design (RCT, case-control, pre-post-introduction) and number of vaccine doses for outcomes, and pooled estimates with more than one study available in a stratum. Meta-analysis of effect measures was conducted using the DerSimonian-Laird pooled risk ratio to provide conservative estimates in the presence of heterogeneity and presented with a 95% confidence interval (CI). All analyses were performed using STATA software (version 12.1, Stat Corp, College Station, TX). Sensitivity analyses included pooling studies with exact number and greater number of doses (i.e. combined effects of 1 dose with those of ≥ 1 doses), restricted age groups, multi-year post-introduction time periods (rather than single years), and apparent elimination (i.e. zero events) in the post-introduction time period in time-series studies.

LiST projects the impact of child survival interventions on syndrome-specific causes of child mortality. To provide an effect size estimate for inclusion in LiST, we pooled estimates across multiple studies (≥2) of the same design reporting dose-specific effects for outcomes approximating meningitis mortality, then combined these with meningitis etiology data from existing reviews [17,18] to determine the Hib and PCV vaccine preventable fractions of childhood meningitis mortality. We computed 1,000 Monte Carlo simulations for each parameter given a triangular distribution and uncertainty limits determined by the 95% uncertainty interval for each of the parameters (i.e. proportion of meningitis caused by Hib or PCV, vaccine effectiveness and for PCV the vaccine serotype coverage.) Multiplying these simulated parameters and using the resulting distribution of vaccine attributable fractions, we took the 2.5th and 97.5th percentile as the approximate central 95% uncertainty interval. The estimated values for meningitis etiology, vaccine serotype coverage and vaccine preventable fractions of childhood meningitis mortality are for illustrative purposes and can be modified by LiST users.

## Results

### Studies identified

**Figure 2:**
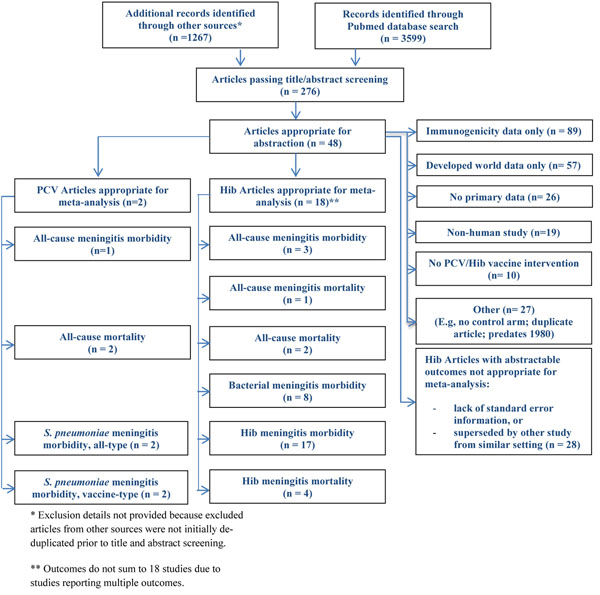
Flow chart of studies reporting the effect of Hib or pneumococcal conjugate vaccines on meningitis outcomes.

The systematic literature review yielded 3599 unique citations from Pubmed and 1267 non-deduplicated citations from other sources (Figure 2), of which 20 contained abstractable data on the effects of Hib conjugate vaccines (*n*=18) and PCVs (*n*=2) in prevention of meningitis outcomes or all-cause mortality in low- and middle-income countries. Eligible citations were frequently excluded in full-text screening due to reporting of overlapping data with another citation, lack of a clearly-defined population for catchment area, or failure to meet analytic criteria due to lack of standard error information [19-45].

### Quality assessment and Hib vaccine effects

The 18 included studies reported the effects of Hib-containing conjugate vaccines including PRP-tetanus toxoid (PRP-T) or PRP-diphtheria toxoid (PRP-CRM), alone or mixed with other antigens. Characteristics and outcomes from included studies are summarized in additional file, Table 3. Note that this table is in the additional file as it was too large to present in the document. Our primary objective was to estimate the effects of Hib conjugate vaccines on childhood meningitis mortality; we identified a single RCT [46] from Indonesia reporting the effects on all-cause meningitis mortality (RR: 0.87, 95% CI: 0.32-2.41), but it cannot be included in LiST as CHERG guidelines require ≥2 studies for an effect. Similarly, a single RCT [46] (RR: 0.11, 95% CI: 0.01-1.17) and a very-low-quality case-control study [47] (RR: 0.29, 95% CI: 0.06-1.37) reported non-statistically significant reductions in risk of Hib meningitis mortality. Also, evidence on the effect of at least one dose of Hib conjugate vaccine on all-cause mortality was available from RCTs in Indonesia [48] and The Gambia [46], yielding a pooled estimate of a 4% reduction in all-cause mortality (RR: 0.96, 95% CI: 0.86-1.08), but since LiST models syndrome-specific intervention effects, this outcome is not recommended for the effect estimate.

More evidence of the effects of Hib conjugate vaccines was available for meningitis morbidity outcomes. Observational studies of the effects of Hib conjugate vaccines on meningitis outcomes included case-control studies of Hib vaccine effectiveness (*n*=6) and time-series analyses comparing outcomes pre- and post-Hib vaccine introduction often using surveillance data (*n*=9). A majority of the case-control studies were from African countries (*n*=4) while most time-series analyses were conducted in Latin America and Caribbean countries (*n*=7). The evidence of the effects of Hib on meningitis outcomes was limited in the Asia (1 RCT, 1 case-control study) and Oceania (1 time-series analysis) regions. Three studies, including a single RCT [48] (RR=0.50, 95% CI: 0.27-0.82) and two pre-post vaccine introduction time-series analyses [49,50] (RR=0.70, 95% CI: 0.54-0.89) estimated the overall effect of Hib conjugate vaccine on incidence of all-cause meningitis. A single RCT [48] and six observational studies (case-control and time-series analyses) [49-54] provided very-low-to-moderate quality evidence on the effect of Hib vaccine on incidence of acute bacterial or probable bacterial meningitis.

**Figure 3:**
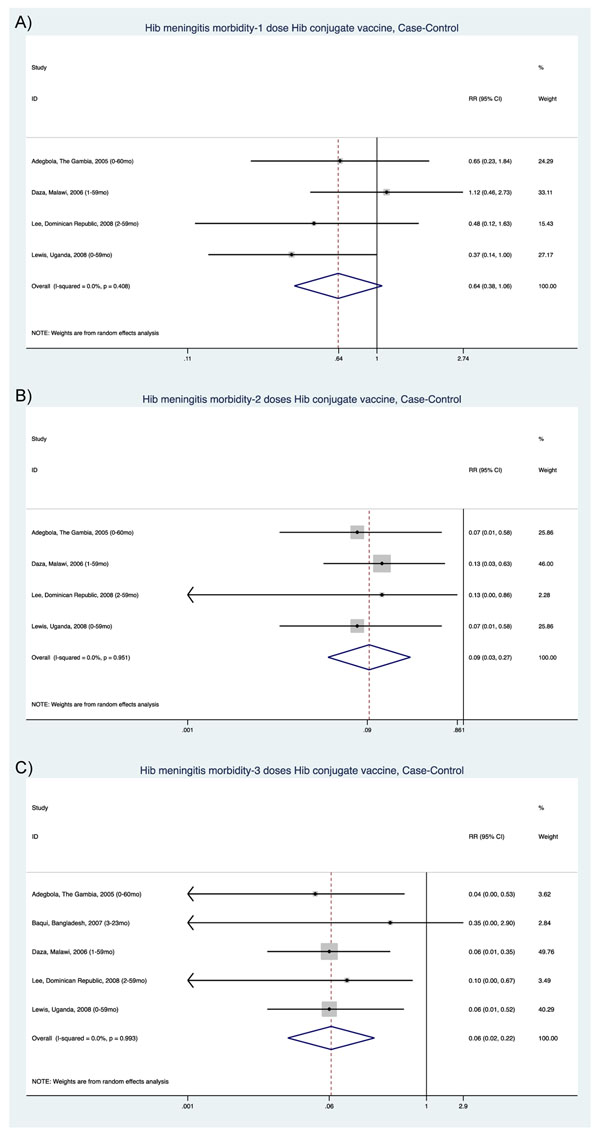
Meta-analysis of observational studies examining the effect of Hib conjugate vaccines on Hib meningitis incidence by: A) 1 dose, B) 2 doses, C) 3 doses of vaccine.

Thus, although our primary interest was to estimate dose-specific effects of Hib conjugate vaccine on meningitis mortality, sufficient evidence was available only for Hib meningitis morbidity outcomes. Thirteen of the observational studies reporting morbidity outcomes [49-61], of very-low-to-moderate quality, estimated effects on Hib meningitis incidence, but dose-specific effects were only available from the subset that were case-control studies (Figure 3). Our meta-analysis of the four pooled case-control studies examined the dose-specific effect of Hib conjugate vaccines on Hib meningitis morbidity (1 dose: RR=0.64, 95% CI 0.38-1.06; 2 doses: RR=0.09, 95% CI 0.03-0.27; 3 doses: RR=0.06, 95% CI 0.02-0.22); and these estimates were consistent with results from three doses of Hib vaccine administered in two RCTs [46,48] (pooled RR: 0.12, 95% CI: 0.03-0.51) and the pooled estimate across time-series studies evaluating pre- vs. post-Hib introduction (RR: 0.02; 95% CI: 0.00-0.15).

### Quality assessment and PCV vaccine effects

**Figure 4:**
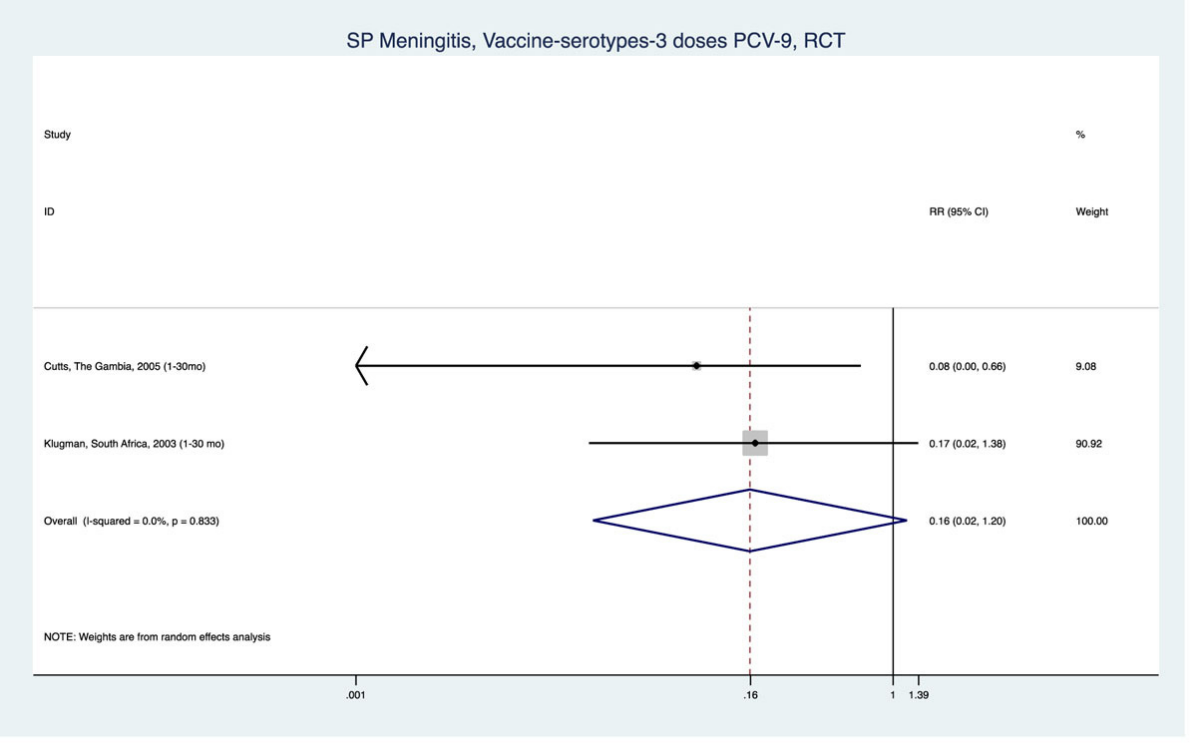
Meta-analysis of randomized controlled trials (RCTs) examining the effect of three doses of pneumococcal conjugate vaccine on incidence of vaccine-type (VT) pneumococcal meningitis.

We identified only two studies, both high-quality RCTs among children age 1-30 months from South Africa [62] and The Gambia [63], reporting the effects of PCV on meningitis-related outcomes. The overall reduction in all-cause mortality associated with at least one dose of the nine-valent vaccine (PCV-9) was 10% across studies. (pooled RR=0.90, 95% CI: 0.80-1.01), but as above, all-cause mortality cannot be used for LiST. An estimate of all-cause meningitis mortality reduction with three doses of PCV-9 was only available from the South African RCT (RR: 0.87, 95% CI: 0.32-2.41). Finally, both RCTs also reported evidence on the effects of three doses of PCV-9 on pneumococcal meningitis morbidity. Although the findings indicate non-statistically significant reductions in incidence of AT pneumococcal meningitis (pooled RR=0.88, 95% CI: 0.47-1.62) and to VT meningitis in particular (pooled RR=0.16, 95% CI: 0.02-1.20), the direction and magnitude of the effects were similar across both RCTs (Table 1, Figure 4).

**Table 1:** Studies of the effect of pneumococcal conjugates vaccines with meningitis outcomes.

		Quality Assessment			Directness			Effect		
No of studies	Doses	Design	Limitations	Consistency	Generalizability to Population of Interest	Generalizability to Intervention of Interest	Age range (months)	Events (control or pre-introduction)	Events (intervention or post-introduction)	Measure **or Meta-estimate**
***All-cause meningitis morbidity: High -quality measure of outcome-specific mortality***										

1 [62]	3 doses	RCT	High quality-None major	Not applicable	moderate-Africa	moderate-1 vaccine (PCV-9)	1-30	8	7	0.87(0.32-2.41)

***All-cause mortality***										

2 [62,63]	1 dose 3 doses (goal)	RCT	High quality-None major	high-evidence of dose-response effect	high-Africa	moderate-1 vaccine (PCV-9)	1-30	389 242	330 229	**0.90(0.80-1.01)**

***SP Meningitis, All-serotypes***										

2 [62,63]	3 doses 3 doses (goal)	RCT	High quality-None major	high-consistent effect magnitude	high-Africa	moderate-1 vaccine (PCV-9)	1-30	17 6	19 5	**0.88(0.47-1.62)**

***SP Meningitis, Vaccine-serotypes***										

2 [62,63]	3 doses 3 doses (goal)	RCT	High quality-None major	moderate-consistent direction of effect	high-Africa	moderate-1 vaccine (PCV-9)	1-30	18 6	2 1	**0.16(0.02-1.20)**

### Effect size estimates for inclusion in LiST

Using the CHERG grading system to provide recommendations on evidence to be used to estimate the effect sizes for inclusion in LiST, we only found sufficient data to estimate dose-specific effects of Hib conjugate vaccines for the Hib meningitis morbidity outcome. For Hib conjugate vaccines, evidence reported for the effect on all-cause mortality, all-cause meningitis mortality, all-cause and acute bacterial meningitis morbidity were available from single or multiple studies of varying study designs and different numbers of doses (e.g. at least 1 dose or 3 doses) but dose-specific evidence was only available for each dose (1, 2, and 3 doses separately) from case-control observational studies evaluating the effects on Hib meningitis incidence.

Ideally, dose-specific effects should be estimated using the same studies for each dose number, to reduce the probability that the differential impact observed between doses is due to inclusion of different studies rather than actual differences between doses. We identified four case-control studies reporting the dose-specific effects of Hib conjugate vaccine on incidence of Hib meningitis, performed sensitivity analyses including evidence from other studies for which all dose-specific effects were not available, and retained these other studies in the pooled estimates if inclusion resulted in little or no impact on the effect measure. This measure of the protective effect of Hib conjugate vaccines on incidence of Hib meningitis was assumed to approximate the effects on Hib meningitis mortality.

Since LiST models the effects of interventions on *syndrome-specific* child mortality, we then combined the pooled effects on Hib meningitis with meningitis etiology data (i.e. proportion of meningitis due to Hib) to determine the preventable fraction of childhood meningitis mortality for inclusion in LiST as seen in Table 2. We used meningitis etiology data from an existing CHERG review [64] for illustrative purposes; the value can be re-calculated with other source data and entered by the LiST user. These combined data provide an estimated dose-specific effect of Hib conjugate vaccines on childhood meningitis mortality in low-and middle-income countries ranging from a 14%, 36% and 38% reduction in a meningitis belt setting with 1, 2 and 3 doses, respectively, and 17%, 42% and 43% reduction elsewhere.

**Table 2:** Effect size estimates of Hib conjugate vaccines on childhood meningitis mortality for inclusion in LiST. (A) Approximated using evidence from existing reviews and can be edited by the LiST user. (B) Estimate of the effectiveness of Hib vaccines against Hib meningitis mortality (risk ratio) from this review. (C) Effect size for Hib conjugate vaccines on meningitis mortality for LiST calculated by multiplying (A) x (B). (D) Uncertainty bounds. †Effectiveness of Hib vaccines against Hib meningitis mortality is approximated by incidence of Hib meningitis disease.

	Proportion of all-cause meningitis mortality due to Hib [1] (A)	Effectiveness of Hib vaccines against Hib meningitis mortality (risk ratio)†(B)	Effect size for Hib vaccine on meningitis mortality (A x B) = (C)	Uncertainty bounds (D)
African meningitis belt				

1 dose	0.40 (0.30-0.52)	0.36 (0.0-0.62)	0.14	0.03-0.20
2 doses	0.40 (0.30-0.52)	0.91 (0.73-0.97)	0.36	0.27-0.41
3 doses	0.40 (0.30-0.52)	0.94 (0.78-0.98)	0.38	0.29-0.42

Other regions				

1 dose	0.46 (0.40-0.54)	0.36 (0.0-0.62)	0.17	0.03-0.22
2 doses	0.46 (0.40-0.54)	0.91 (0.73-0.97)	0.42	0.34-0.45
3 doses	0.46 (0.40-0.54)	0.94 (0.78-0.98)	0.43	0.36-0.46

With respect to PCVs, two high-quality RCTs reported the effects of PCV-9 on both all-cause mortality and pneumococcal meningitis in low-income countries. As above, because LiST does not model the effect of childhood interventions on all-cause mortality, we used the pooled estimate of the effects of 3 doses of PCV-9 against vaccine-type pneumococcal meningitis, combined with meningitis etiology data (proportion of meningitis due to pneumococcus) and vaccine serotype coverage (proportion of pneumococcal meningitis caused by vaccine serotypes approximated from invasive pneumococcal disease) from existing reviews [65,66] to provide an estimated effect of PCV on childhood meningitis mortality in low-and middle-income countries. Applying this approach to an example using the existing 10-valent PCV (PCV-10), we estimated region-specific effects ranging from 28-35% reduction in childhood meningitis mortality with 3 doses of PCV-10 (Table 3). Use of the existing thirteen-valent PCV (PCV-13) would likely increase the effect size.

**Table 3:** Effect size estimates of pneumococcal (SP) conjugate vaccines (PCV) on childhood meningitis mortality for inclusion in LiST. (A) Approximated using evidence from existing reviews and can be edited by the LiST user [1]. (B) Approximated from the proportion of invasive pneumococcal disease caused by vaccine serotypes in the existing 10-valent PCV from an existing review and can be edited by the LiST user [17]. (C) Estimate of the effectiveness of PCV against vaccine-serotype SP meningitis mortality (risk ratio) from this review. (D) Effect size for PCV on meningitis mortality for LiST calculated by multiplying (A) x (B) x (C). (E) Uncertainty bounds.

	Proportion of all-cause meningitis mortality due to SP [1,18] (A)	Proportion of SP meningitis caused by vaccine serotypes (B)	Effectiveness of PCV against vaccine-type SP meningitis mortality (C)	Effect size for PCV on meningitis mortality (A x B x C) = (D)	Uncertainty bounds (E)
African meningitis belt	0.46 (0.42-0.50)	0.72 (0.67-0.76)	0.84 (0.00-0.98)	0.28	0.05-0.30
Africa non-meningitis belt	0.52 (0.47-0.58)	0.72 (0.67-0.76)	0.84 (0.00-0.98)	0.32	0.05-0.34
Asia	0.52 (0.47-0.58)	0.70 (0.64-0.75)	0.84 (0.00-0.98)	0.31	0.05-0.33
Europe	0.52 (0.47-0.58)	0.81 (0.78-0.82)	0.84 (0.00-0.98)	0.35	0.06-0.38
Latin America and Caribbean	0.52 (0.47-0.58)	0.77 (0.74-0.80)	0.84 (0.00-0.98)	0.34	0.06-0.36
Oceania	0.52 (0.47-0.58)	0.75 (0.63-0.84)	0.84 (0.00-0.98)	0.33	0.06-0.35

## Discussion

Lack of dose-specific estimates on the effects of Hib and PCV on childhood meningitis mortality from high-quality studies (e.g. RCTs) complicated the quantification of effect sizes to be incorporated into LiST. Additionally, existing CHERG guidelines are more easily applied to interventions with a single coverage stratum (e.g. coverage with 3 doses of vaccine) rather than dose-specific effects (e.g. coverage with 1, 2 and 3 vaccine doses) which require evidence for the effect of each dose across multiple studies.

Despite the limited number of RCTs evaluating the effects of Hib conjugate vaccine on childhood meningitis mortality, we identified considerable evidence from observational studies, both case-control and time-series pre- and post-vaccine introduction, and across a range of meningitis outcomes, with relative consistency observed across study designs and settings. We also performed a series of sensitivity analyses including studies with restricted age ranges and different numbers of vaccine doses (e.g. effects of exactly 3 doses combined with those of 3 or more doses), and the effect on incidence of Hib meningitis appeared robust in sensitivity analyses.

For comparison, we assessed our findings with those from two recent global reviews measuring the efficacy of Hib vaccine against invasive Hib disease in a meta-analysis of RCTs by Griffiths et al. [67], and a review of observational studies by Jackson et al.[68] Among studies from developing countries [69] we included in our analyses the same studies with the exception of those lacking either clearly defined populations or sufficient information to calculate standard error.^31^[70] Despite these slight differences in included studies, and in specificity of outcome of interest, these two reviews reported pooled estimates of Hib conjugate vaccine efficacy against invasive Hib disease consistent with our estimates. We identified limited data available for PCV from observational studies or other reviews to allow for a similar assessment of the consistency of our results, but this will be possible when PCV impact assessments from early adopting low-income countries become available in the near future.

These estimates, as may be true of LiST estimates for some other vaccines, probably significantly underestimate the population-wide impact of the interventions. The impact measures used for the LiST estimates provided here reflect the indirect or herd effect minimally if at all (as they are based on case-control studies and RCTs) and LiST does not currently incorporate indirect effects into projections. However, its importance is well-documented in empirical studies in developed countries [71-76], and would be expected to be significant for populations both within and beyond the age groups modeled in LiST.

Several other limitations may affect these estimates as well, including the quality and comparability of studies in the analysis and the assumptions required to quantify an effect size for inclusion in LiST. Higher-quality data was available for some Hib outcomes but these either were from single studies or did not provide dose-specific effect estimates. While there was some data from most regions, there was not sufficient data to provide region-specific estimates of the effects of Hib or PCV vaccines on meningitis. The generalizability of the two PCV studies, particularly for the South African trial limited to hospitalized children, is not clear. Incomplete documentation of vaccination status in case-control studies, and of immunization coverage for time-series impact assessments, could also have biased results. Indirect effects may provide an important contribution to vaccine impact, but no data were available for PCV and LiST does not currently model the impact of indirect effects of interventions. Future versions of LiST may be able to incorporate estimates of both direct and indirect effects of interventions.

In addition to the limitations of the vaccine efficacy or effectiveness data, the assumptions and approximations required to be able to quantify the dose-specific effects of Hib and PCV vaccines provide additional uncertainty in the results. First, we assumed that vaccine effects on incidence of Hib meningitis and vaccine-type pneumococcal meningitis approximate effects on organism-specific childhood meningitis mortality. Bacterial meningitis typically causes severe disease and with a high case fatality ratio, therefore this assumption is plausible. Second, we used meningitis etiology data from an existing review [77] to estimate the proportion of meningitis due to Hib or pneumococcus. Third, for PCV, we assumed that vaccine coverage of serotypes causing invasive pneumococcal disease from a recent review [78] was equivalent to coverage of serotypes causing pneumococcal meningitis mortality; this may not be completely accurate, since most IPD cases are due to bacteremic pneumonia, and syndrome-specific serotype differences between syndromes have been reported [79]. The assumed values of vaccine-preventable fractions of childhood meningitis mortality were for illustrative purposes using available evidence, and can be changed by the LiST user if higher-quality or more representative data are available for individual country projections. The multiplication of multiple parameters to estimate the vaccine-preventable fraction of meningitis mortality increases the degree of uncertainty. However, we are encouraged by the consistency of our findings with results from single RCTs and observational studies from other similar reviews. The results of this analysis suggest that 3 doses of Hib and PCV together would prevent approximately three-quarters of childhood meningitis mortality, leaving a remaining one-quarter caused by non-vaccine type pneumococcal meningitis, meningococcal meningitis and other causes of meningitis/encephalitis.

## Conclusions

Estimates of the dose-specific effectiveness of PCVs and Hib conjugate vaccines in preventing childhood meningitis mortality are limited by sparse data that met the criteria for our meta-analysis, including high quality prospective studies in low income settings. Despite these limitations, available evidence on impact of these vaccines on meningitis morbidity indicates that a majority of childhood meningitis mortality is preventable with existing Hib and PCV vaccines, and these findings are consistent with other empirical evidence and reviews. Our understanding of the impact of PCV in low-income settings is likely to significantly improve over the next several years as results from early PCV adopting countries are made publicly available. Inclusion of dose-specific and indirect effects of interventions will likely improve the accuracy of LiST country impact projections.

## Key messages

Available evidence indicates that a majority of childhood meningitis mortality is preventable with existing Hib and PCV vaccines and these findings are consistent with other empirical evidence and reviews. This review quantified the effects of Hib and pneumococcal conjugate vaccines and will be incorporated into the Lives Saved Tool (LiST) to model the projected impact on childhood meningitis mortality in low income countries.

## List of abbreviations used

AT: all-seroypes of pneumococcus; CHERG: Child Health Epidemiology Reference Group; CI: confidence interval; Hib: *Haemophilus influenzae* type b; LiST: Lives Saved Tool; PCV: pneumococcal conjugate vaccine; RCT: randomized controlled trial; RR: risk ratio; VT: vaccine-containing serotypes of pneumococcus.

## Competing interests

None declared.

## Authors’ contributions

SD supervised the abstraction and drafted and edited the manuscript. DF edited the manuscript. HJ conceived of the study, performed all analyses, and edited the manuscript. All authors read and approved the final manuscript.

## Supplementary Material

Additional File 1 **Supplementary Tables 1,2 and 3**. Supplementary Table 1 (sheet 1): Complete data on included Hib studies. Supplementary Table 2 (sheet 2): Compete data on included PCV studies. Supplemental Table 3: Data from final Hib studies used in effect size estimation. Click here for file

## Declarations

The publication costs for this supplement were funded by a grant from the Bill & Melinda Gates Foundation to the US Fund for UNICEF (grant 43386 to "Promote evidence-based decision making in designing maternal, neonatal, and child health interventions in low- and middle-income countries”). The Supplement Editor is the principal investigator and lead in the development of the Lives Saved Tool (LiST), supported by grant 43386. He declares that he has no competing interests.

This article has been published as part of *BMC Public Health* Volume 13 Supplement 3, 2013: The Lives Saved Tool in 2013: new capabilities and applications. The full contents of the supplement are available online at http://www.biomedcentral.com/bmcpublichealth/supplements/13/S3.
